# The *Listeria monocytogenes* exopolysaccharide significantly enhances colonization and survival on fresh produce

**DOI:** 10.3389/fmicb.2023.1126940

**Published:** 2023-04-27

**Authors:** Alex M. Fulano, Ahmed M. Elbakush, Li-Hong Chen, Mark Gomelsky

**Affiliations:** ^1^Department of Molecular Biology, University of Wyoming, Laramie, WY, United States; ^2^Faculty of Veterinary Medicine, Tripoli University, Tripoli, Libya

**Keywords:** foodborne pathogen, listeriosis, food safety, c-di-GMP, desiccation, acid stress, biofilm, vegetable and fruit

## Abstract

Fresh produce contaminated with *Listeria monocytogenes* has caused major listeriosis outbreaks in the last decades. Our knowledge about components of the listerial biofilms formed on fresh produce and their roles in causing foodborne illness remains incomplete. Here, we investigated, for the first time, the role of the listerial Pss exopolysaccharide (EPS) in plant surface colonization and stress tolerance. Pss is the main component of *L. monocytogenes* biofilms synthesized at elevated levels of the second messenger c-di-GMP. We developed a new biofilm model, whereby *L. monocytogenes* EGD-e and its derivatives are grown in the liquid minimal medium in the presence of pieces of wood or fresh produce. After 48-h incubation, the numbers of colony forming units of the Pss-synthesizing strain on pieces of wood, cantaloupe, celery and mixed salads were 2−12-fold higher, compared to the wild-type strain. Colonization of manmade materials, metals and plastics, was largely unaffected by the presence of Pss. The biofilms formed by the EPS-synthesizing strain on cantaloupe rind were 6−16-fold more tolerant of desiccation, which resembles conditions of whole cantaloupe storage and transportation. Further, listeria in the EPS-biofilms survived exposure to low pH, a condition encountered by bacteria on the contaminated produce during passage through the stomach, by 11−116-fold better than the wild-type strain. We surmise that *L. monocytogenes* strains synthesizing Pss EPS have an enormous, 10^2^−10^4^-fold, advantage over the non-synthesizing strains in colonizing fresh produce, surviving during storage and reaching small intestines of consumers where they may cause disease. The magnitude of the EPS effect calls for better understanding of factors inducing Pss synthesis and suggests that prevention of listerial EPS-biofilms may significantly enhance fresh produce safety.

## Introduction

*Listeria monocytogenes* is one of the most notorious foodborne pathogens. Listeriosis, a severe systemic illness caused by this bacterium, affects primarily the elderly, pregnant women, newborns, and the immune compromised individuals. While the number of listeriosis cases is relatively modest, e.g., approximately 1,600 cases per annum in the USA, mortality rates are extremely high, 15−20% (Centers for Disease Control and Prevention, 2022). This justifies the “zero *L. monocytogenes* tolerance” policy of the US Department of Agriculture (Archer, 2018), which means that large shipments of ready-to-eat food products that are contaminated or suspected to be contaminated with *L. monocytogenes* are often recalled. The high socioeconomic costs associated with contaminated food make *L. monocytogenes* the third “most expensive” pathogen in the USA among all bacterial, viral, and eukaryotic foodborne pathogens (Hoffmann and Ahn, 2021).

While traditional sources of listerial contamination have included ready-to-eat meat, poultry, seafood, and dairy products, the number of listeriosis outbreaks associated with contaminated fresh produce has been growing (Zhu et al., 2017). Major outbreaks in the recent years have been caused by contaminated cantaloupes (also known as rock melons), celery, packaged salads, bean sprouts, caramelized apples, and frozen vegetables (Centers for Disease Control and Prevention, 2022; NSW Government). Among these outbreaks, the 2011 cantaloupe-associated outbreak stands out. It affected people in 28 states resulting in 33 fatalities and one miscarriage, making it one of the highest mortality food-poisoning incidents in the modern US history. That outbreak involved whole cantaloupes contaminated with *L. monocytogenes* at a Colorado cantaloupe processing facility (McCollum et al., 2013). Currently, we poorly understand what factors may have contributed to *L. monocytogenes* colonization of the whole cantaloupes, and helped bacteria survive on the inhospitable surface of cantaloupe rind during extended periods of storage and transportation throughout the USA. This situation applies to other kinds of fresh produce, which impedes our ability to prevent fresh produce-associated listeriosis outbreaks (Marik et al., 2020).

On the surfaces of various materials bacteria grow in biofilms, where aggregated cells are surrounded by the secreted extracellular matrix that enhances their survival (Costerton et al., 1999; Flemming and Wingender, 2010; Flemming et al., 2016). In the environment, listeria, which is frequently associated with the decaying plant matter (Welshimer and Donker-Voet, 1971; Fenlon, 1986; Ivanek et al., 2007; Freitag, 2009), likely grows in biofilms as well, yet we do not know composition of listerial biofilms in the environment. The studies of plant-associated biofilms in various other bacteria revealed that the extracellular biofilm matrix comprises exopolysaccharide (EPS), extracellular DNA (eDNA), fimbriae/pili and amyloid fibers (Flemming and Wingender, 2010; Limoli et al., 2015; Karygianni et al., 2020). Fimbriae/pili and amyloid fibers were never observed in *L. monocytogenes*. eDNA is an abundant component of listerial biofilms on various materials (Harmsen et al., 2010; Zetzmann et al., 2015). The role of EPS as component of plant-associated listerial biofilms has not been studied, until this work. The presence of EPS in biofilms formed by various listerial strains was inferred via indirect methods (Borucki et al., 2003; Hefford et al., 2005; Combrouse et al., 2013; Tiensuu et al., 2013), yet until recently, the ability of *L. monocytogenes* to synthesize EPS has remained controversial (Renier et al., 2011).

Our earlier work (Chen et al., 2014) uncovered the ability of *L. monocytogenes* to synthesize EPS, designated Pss, when cellular levels of the second messenger, cyclic dimeric GMP (c-di-GMP), are elevated. The Pss EPS is a unique polymer composed of the repeating trisaccharide unit, {4)-β-ManpNAc-(1−4)-[α-Galp-(1−6)]-β-ManpNAc-(1-}, and is the only cell-attached, insoluble, EPS synthesized by listeria (Köseoğlu et al., 2015). In liquid media, Pss causes cell aggregation, clumping but does not promote attachment to glass or polystyrene materials (Chen et al., 2014). The second messenger c-di-GMP, that activates Pss synthesis, controls EPS synthesis and production of other biofilm components not only in listeria, but in a variety of bacteria (Römling et al., 2013). At present, environmental factors that turn on c-di-GMP synthesis in *L. monocytogenes* are unknown, therefore, Pss synthesis is induced by genetic means, e.g., by expressing a heterologous c-di-GMP synthase, diguanylate cyclase (DGC), or by inactivating c-di-GMP phosphodiesterases (PDEs) that hydrolyze c-di-GMP (Chen et al., 2014). The rise in cellular c-di-GMP levels is conveyed to the Pss biosynthetic apparatus by the c-di-GMP-effector protein, PssE, whose gene is encoded within the *pssA-E* biosynthesis operon (Chen et al., 2014).

To investigate the role of Pss in *L. monocytogenes* colonization of plant surfaces, we developed a listeria-plant biofilm model that involves incubation of the autoclaved wood coupons (disks), as substitutes for partially degraded wood present in the environment, in the minimal medium inoculated with listeria. We also used pieces of fresh produce in place of wood coupons. The plant material was incubated in liquid medium to allow not only bacterial attachment but also biofilm formation. We used the EGD-e wild-type strain and its derivatives that express varying EPS levels. Strain Δ*pdeB/C/D* constitutively expresses EPS because it lacks c-di-GMP-specific PDEs, PdeB, PdeC and PdeD (Chen et al., 2014). Strain Δ*pdeB/C/D*Δ*pssC* contains elevated c-di-GMP levels but carries a mutation in the gene encoding the key Pss biosynthetic enzyme, glycosyltransferase PssC, which impairs Pss synthesis (Köseoğlu et al., 2015). Yet another strain, engineered in this work, lacks all DGCs and PDEs, Δ*pdeB/C/D ΔdgcA/B/C*, is devoid of c-di-GMP and therefore, impaired in Pss synthesis.

We found that the Pss EPS promotes *L. monocytogenes* colonization of wood, cantaloupe rind and pulp, celery, and lettuce, but does not affect colonization of abiotic, manmade materials. We also found that listeria growing in the EPS-biofilms on plant surfaces are much more tolerant of desiccation and acid stress. The enhanced colonization of plant matter, increased tolerance of desiccation and acid stress (conditions that mimic, respectively, fresh produce storage and exposure to stomach acid following produce consumption), may give *L. monocytogenes* EPS-biofilms enormous advantage in causing foodborne illness. Results of this work imply that *L. monocytogenes* EPS-biofilms may present a serious problem for fresh produce industry.

## Results

### EPS strongly promotes *L. monocytogenes* colonization of the surfaces of wood but not manmade materials

To study listerial biofilms on plant surfaces, we departed from the commonly used models. First, instead of growing *L. monocytogenes* in rich media that contains peptides, amino acids, lipids, and nucleotides or nucleic acids, we switched to minimal medium containing glucose as carbon source, because such medium better approximates conditions under which listeria interact with plant matter in nature and in fresh produce processing facilities. Second, we incubated autoclaved wood coupons (as substrates mimicking partially degraded wood in the environment) and sterilized pieces of fresh produce in the liquid medium inoculated with *L. monocytogenes* for extended period, 48 h, to allow biofilm formation.

**FIGURE 1:**
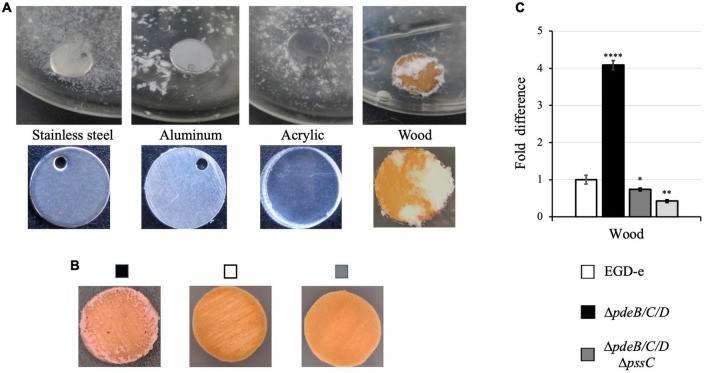
The listerial Pss EPS enhances colonization of wood but not manmade materials. *L. monocytogenes* strains were grown in liquid HTM/GY medium at 30°C for 48 h in the presence of coupons (disks) made from wood and manmade materials. **(A)** The EPS-synthesizing strain (Δ*pdeB/C/D*) grown in the presence of coupons made from stainless steel, aluminum and acrylic, and (unknown tree) wood forms voluminous biofilms only on wood coupons. **(B)** The EPS-synthesizing strain, but not wild type or the EPS-impaired strain (Δ*pdeB/C/D*Δ*pssC*), form biofilms on wood coupons. All strains express red fluorescent protein mScarlet, which turns biomass pink (Table 1; Bindels et al., 2017). **(C)** Comparison of the CFU numbers in biofilms formed by various strains on the wood coupons following removal of the loosely attached biomass by rinsing. Shown are fold-differences compared to the wild type (EGD-e). The average CFU numbers of the wild type were 8.0 × 10^8^ CFU per coupon. Data are from three independent experiments with at least two replicates in each experiment. Error bars represent standard deviations. Significant differences, compared to EGD-e, are indicated as follows: *P < 0.05, ^**^P < 0.01, and ^****^P < 0.0001.

Following incubation, a significant fraction of biomass of the Pss EPS-synthesizing Δ*pdeB/C/D* strain was attached to the wood coupons (Figures 1A, B). This contrasted poor colonization of surfaces of the manmade materials, i.e., stainless steel, aluminum or acrylic (Figures 1A, B). This finding is consistent with our earlier observations that listerial EPS-aggregates formed in liquid medium poorly attach to polystyrene and glass surfaces (Chen et al., 2014). To test if surface roughness, as opposed to the nature of the material, played a major role in attachment, we scratched the surfaces of manmade materials thus bringing surfaces roughness closer to the roughness of the wood coupons. This did not improve bacterial attachment (not shown), suggesting that EPS-synthesizing listeria strongly prefer wood over manmade materials.

In contrast to the voluminous biofilms formed on wood coupons by the Pss-synthesizing strain, 48-h old biofilms of the wild-type strain, EGD-e, or the Δ*pdeB/C/D*Δ*pssC* mutant impaired in EPS synthesis were not visible to the naked eye (Figures 1A, B). To quantify attached cells, the biofilm biomass was scraped off from the surface of wood coupons, vigorously vortexed in liquid medium, and serial dilutions were plated for enumerating colony forming units (CFUs). Figure 1C shows that the Pss EPS strongly, by at least fourfold, promotes colonization of wood coupons. Because prior to biomass scraping, loosely associated aggregates were removed by rinsing in sterile medium, the reported fold-difference is an underestimate.

**FIGURE 2:**
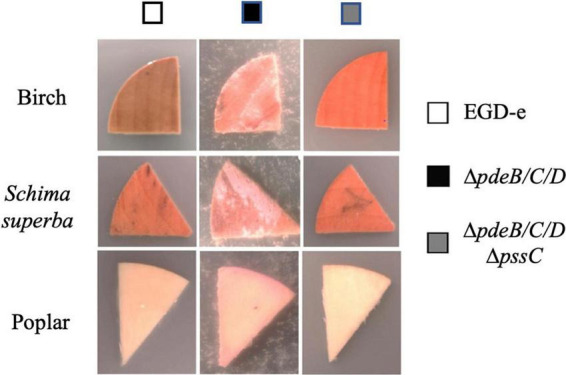
The listerial EPS-enhanced colonization of wood from various tree species. Coupon pieces from three tree species (birch, *Schima superba* and poplar) show higher colonization by the *L. monocytogenes* EPS-synthesizing strain (Δ*pdeB/C/D*), compared to the wild type (EGD-e) and EPS-impaired strain (Δ*pdeB/C/D*Δ*pssC*). The pink color of the biomass is due to the expressed mScarlet protein. Wood pieces were incubated in the HTM/GY minimal medium in the presence of listeria for 48 h at 30°C.

To assess whether preference for wood is specific to the (unknown) tree species from which coupons were made, we tested coupons made from three known tree types. The EPS-synthesizing strain showed improved attachment to all tested wood coupons, albeit to varying extent (Figure 2). Therefore, listerial Pss increases attachment to various kinds of wood.

### The Pss EPS increases *L. monocytogenes* colonization of fresh produce

Next, we investigated the role of the Pss EPS in listerial colonization of fresh produce using produce types that were associated with the past listeriosis outbreaks. We tested the role of Pss in biofilm formation on cantaloupe rind, the cause of major listeriosis outbreaks in the US and Australia (McCollum et al., 2013; NSW Government, 2018), as well as on cut pieces of fresh produce, which caused the majority of listeriosis outbreaks (Marik et al., 2020; Centers for Disease Control and Prevention, 2022). Sterilized pieces of approximately the same sizes were inoculated with *L. monocytogenes* in flasks containing minimal medium at 30°C.

**FIGURE 3:**
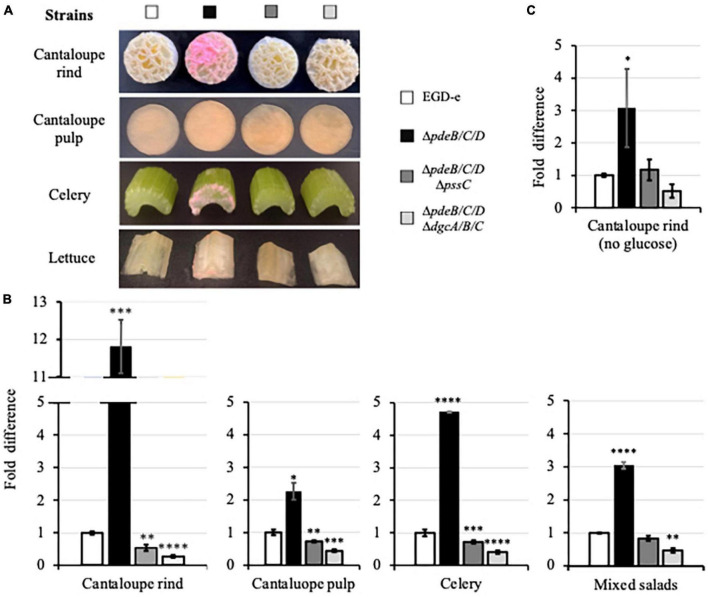
The EPS-enhanced colonization of the pieces of fresh produce. **(A)** Pieces of cantaloupe (containing or lacking rind), celery and Iceberg lettuce (a component of mixed salad) following incubation in the HTM/GY medium with *L. monocytogenes* for 48 h at 30°C. The biomass appears pink due to the expressed mScarlet protein. **(B)** Comparison of the CFUs of *L. monocytogenes* in biofilms formed on the fresh produce pieces incubated in the HTM/GY medium. Shown are fold-differences compared to the wild type (EGD-e). The average CFU numbers for the wild type were 7.0 × 10^9^ CFU per cantaloupe rind, 1.2 × 10^10^ CFU per cantaloupe pulp, 1.9 × 10^8^ CFU per piece of celery, and 8.0 × 10^8^ CFU per three pieces (lettuce, carrot, cabbage) of mixed salad. Error bars represent standard deviations. **(C)** Comparison of the *L. monocytogenes* CFUs in biofilms formed on cantaloupe rind in the HTM medium lacking glucose and yeast extract. The average CFUs of the wild type was 7.4 × 10^6^ CFU per cantaloupe rind. In all figures, data are from three independent experiments with at least two replicates in each experiment. Standard deviations are shown on the graph as error bars. Significant differences, compared to the wild type, are indicated as follows: *P < 0.05; **P < 0.01; ***P < 0.001, and ****P < 0.0001.

After 48-h incubation, the EPS-synthesizing strain formed visible biofilms on cantaloupe rind (Figure 3A). Such biofilms were also visible on the surfaces of celery and lettuce (Figure 3A), most prominently on the cutting edges. No biofilms were visible on the pieces of produce grown in the presence of the wild-type and EPS-impaired strains (Figure 3A). Biofilms of the EPS-synthesizing strain contained ∼2−12-fold higher CFUs, compared to the wild type, and ∼4−22-fold higher, compared to the EPS-impaired strain (Figure 3B), suggesting that EPS strongly promotes colonization of various plant surfaces. The effect of EPS on colonization of cantaloupe rind versus pulp was stronger (12-fold versus 2-fold) (Figure 3B), suggesting that plant surface composition and roughness play important roles in EPS-dependent *L. monocytogenes* colonization.

In the experiments described above, minimal media contained glucose as a carbon source. To mimic conditions at cantaloupe storage and processing facilities, where water/moisture may be present, but carbon source is likely absent or limiting, we repeated the experiment using minimal medium lacking glucose. Residual bacterial growth under those conditions was possible due to the juice leaking from cantaloupe pieces. The lack of glucose in the medium significantly, by almost 10^3^-fold, decreased colonization of cantaloupe rind (7.4 × 10^6^ versus 7.0 × 10^9^ CFU, Figures 3B, C). However, the trend in surface colonization was preserved, i.e., the EPS-synthesizing strain colonized cantaloupe rind several-fold more efficiently than the wild-type or the EPS-impaired strains (Figure 3C).

**FIGURE 4:**
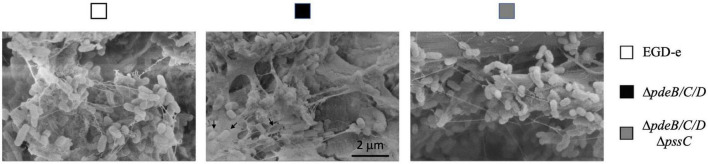
The SEM images of *L. monocytogenes* biofilms formed on cantaloupe rind. Bacterial cells embedded in the EPS matrix and presumed empty shells around bacteria removed during sample preparation (black arrows) are seen in the EPS-synthesizing strain but not in the wild type and EPS-impaired strains. Magnification, 40,000×.

For a closer look at the biofilms on cantaloupe rind, we employed scanning electron microscopy (SEM). All strains formed extracellular appendages on cantaloupe rind, which may represent a combination of eDNA, nanotubes and flagella (Figure 4) and will not be discussed further. The biofilms formed by the EPS-synthesizing strain showed abundant EPS matrix in which listeria were embedded (Figure 4). We were unable to unambiguously determine if the wild type formed any Pss. It showed somewhat higher attachment to plant matter, compared to the EPS-impaired strain, Δ*pdeB/C/D*Δ*pssC* (Figures 1C, 3B), however, whether this was due to low levels of Pss is unclear.

### The EPS-biofilms enhance *L. monocytogenes* tolerance to desiccation

Earlier we showed that the Pss EPS enhances *L. monocytogenes* tolerance to desiccation, when cell aggregates grown in liquid medium were compared to planktonically grown cells (Chen et al., 2014). Desiccation is the condition experienced by listeria during storage of fresh produce, such as whole cantaloupes, at the processing facilities and transportation to supermarkets and consumer homes and restaurants (Esbelin et al., 2018). To assess the role of the Pss EPS-biofilms on fresh produce surfaces in desiccation tolerance, we subjected the 48-h old biofilms formed on cantaloupe rind to drying on air at room temperature for up to 3 weeks. For ease of comparisons, we adjusted CFUs of surface-attached bacteria of all strains to approximately the same initial values by mechanically removing ∼90% biofilm of the EPS-synthesizing strain (Figure 3B) prior to the desiccation experiment. We realize that much thinner EPS-biofilms likely resulted in our underestimating the magnitude of desiccation tolerance benefits rendered by the Pss EPS.

**FIGURE 5:**
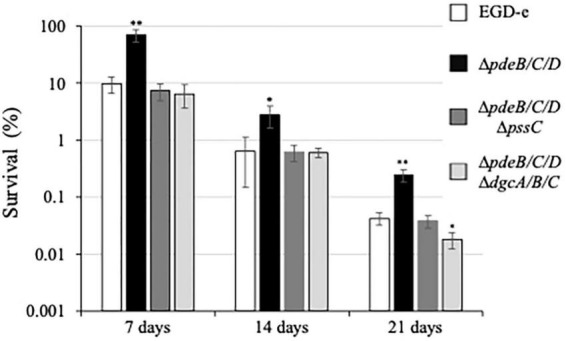
Survival under desiccation of the *L. monocytogenes* biofilms. The 48-h old biofilms formed on cantaloupe coupons containing rind and pulp (diameter, 20 mm; depth 4 mm) were placed in Petri dishes rind up and allowed to dry on open air. A coupon was cut into four quarters. One quarter was used for enumeration of initial CFUs, other quarters were subjected to desiccation and withdrawn on days 7, 14, and 21 days, respectively. Following withdrawal, the coupon quarters were rehydrated, homogenized, and subjected to CFU enumeration. For each strain, survival was calculated based on the initial CFU number of that strain on day 0 (assigned to 100%). Prior to the experiment, ∼90% of the biofilm biomass of the EPS-synthesizing strain, Δ*pdeB/C/D*, was removed from rind to bring the initial CFU numbers close to those of other strains. The average initial CFUs per coupon quarter for all strains were within the range of (1.1–3.4) × 10^8^. Significant differences, compared to EGD-e, are indicated as follows: *P < 0.05 and ^**^P < 0.01.

After 7 days, CFUs decreased in the wild type from 100 to ∼10%, in the EPS-impaired strain to ∼7.4%, and in the EPS-synthesizing strain to 74%, i.e., by ∼10, ∼13, and 1.35-fold, respectively, (Figure 5). During prolonged desiccation, the EPS-synthesizing strain continued to maintain significant survival advantage over other strains (Figure 5). Desiccation tolerance in this strain was 6−16-fold higher, compared to the wild type, over the whole 3 week time course. This implies that the listerial EPS-biofilms formed on fresh produce could survive storage and transportation of fresh produce much better than biofilms formed by the strains not synthesizing Pss.

### The EPS-biofilms enhance *L. monocytogenes* tolerance of acid stress

Prior to reaching hospitable small intestine, the site of the gastrointestinal tract where *L. monocytogenes* causes disease, it faces the challenge of stomach (gastric) acid. To mimic the process of consumption of the contaminated produce, we tested acid stress survival of the listerial biofilms formed on the cut fresh produce. For this experiment, we switched from cantaloupe rind as a model to cantaloupe pulp and celery, because rind is not consumed. The pieces of pulp and celery containing 48-h old listerial biofilms were exposed to acid stress, approximating pH of the stomach acid, i.e., pH 1.5−3.5 (Marieb and Hoehn, 2018). One stress regimen involved a 15-min exposure of the produce pieces to pH 2.5. Another regimen involved a 15-min exposure to pH 5.0, followed by 15 min at pH 2.5 (Guerreiro et al., 2022).

**FIGURE 6:**
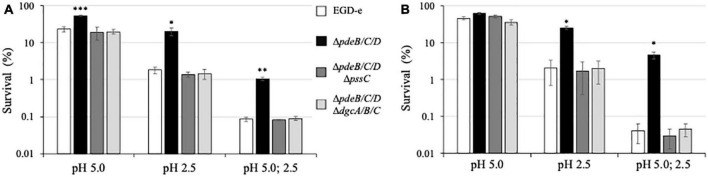
Survival under acid stress of the *L. monocytogenes* biofilms. The 48-h old biofilms formed on pieces of cantaloupe pulp **(A)** and celery **(B)** were exposed for 15 min to pH 5.0 or pH 2.5, or sequentially to pH 5.0 (15 min) and pH 2.5 (15 min). Following medium neutralization, cantaloupe and celery pieces were collected, homogenized, and CFU were enumerated. For each strain, survival percentages were calculated based on the initial CFU prior to exposure to acid (assigned to 100%). The average initial CFUs for all strains were within the range of (5.3–7.3) × 10^9^ per cantaloupe pulp piece and (1.1–1.4) × 10^9^ per celery piece. Significant differences, compared to EGD-e, are indicated as follows: *P < 0.05, ^**^P < 0.01, and ^***^P < 0.001.

As shown in Figures 6A, B, exposure to pH 5 alone resulted in only moderate decreases in bacterial survival in all strains. However, exposure to pH 2.5 uncovered a large difference in survival, i.e., ∼20% for the EPS-synthesizing strain versus ∼1.8% for the wild type and ∼1.4% for the EPS-impaired strain (Figure 6A). Thus gives the EPS-synthesizing strain an ∼11-fold survival advantage over the wild type. Similarly, after a two-step regimen (pH 5.0, then pH 2.5), ∼1% of the EPS-synthesizing strain survived but only ∼0.085% of the wild type and ∼0.081% of the EPS-impaired strain, thus revealing an ∼12-fold advantage of the EPS-synthesizing strain over the wild type. Similar results were obtained when listerial survival in biofilms on the celery pieces was measured (Figure 6B), i.e., ∼25% survival of the EPS-synthesizing strain versus ∼2% for the wild type and ∼1.7% for the EPS-impaired strain following exposure to pH 2.5, and ∼4.7% versus 0.04% and 0.03% survival following a two-step regimen. Thus, the EPS-synthesizing strain has a 12−116-fold survival advantage on celery pieces, compared to the wild-type. These results suggest that from ∼11- to ∼116-fold higher number of *L. monocytogenes* in the EPS-biofilms on cut fresh produce could survive exposure to stomach acid, compared to the biofilms lacking Pss EPS.

### EPS is the primary c-di-GMP-dependent factor responsible for enhanced *L. monocytogenes* plant surface colonization and survival

In *L. monocytogenes*, EPS synthesis is the dominant, albeit not the only phenotype associated with the elevated c-di-GMP levels (Chen et al., 2014; Elbakush et al., 2018). In other bacteria, elevated c-di-GMP levels, in addition to upregulating EPS synthesis, affect such biofilm components as protein adhesins, pili, and fimbria (Römling et al., 2013). To access the impact of c-di-GMP-dependent factors other than Pss EPS that contribute to listerial colonization and survival on plant matter, we constructed the *L. monocytogenes* c-di-GMP null strain, Δ*pdeB/C/D*Δ*dgcA/B/C*. In addition to lacking c-di-GMP-specific PDEs, this mutant also lacks all DGCs involved in c-di-GMP synthesis (Chen et al., 2014). We found that the c-di-GMP null mutant behaved similarly to the high c-di-GMP strain impaired in Pss synthesis, Δ*pdeB/C/D* Δ*pssC*, in survival assays (Figures 5, 6), thus suggesting that Pss is the key factor affecting stress tolerance. In plant surface colonization assays the c-di-GMP null strain appeared somewhat, ∼2-fold, inferior, compared to the Δ*pdeB/C/D* Δ*pssC* strain (Figures 1C, 3B, C). From these experiments, it is evident that the Pss EPS is the dominant c-di-GMP-dependent factor affecting listerial colonization of plant matter and stress tolerance.

## Discussion

In this study, we investigated the role of the Pss EPS in *L. monocytogenes* colonization of plant surfaces. We discovered that Pss significantly, from 2 to 12-fold, improves colonization of diverse plant matter, from different kinds of wood to cantaloupe rind, cantaloupe pulp, celery, and lettuce. Pss has little or no effect on colonization of non-plant, manmade materials (Figures 1–3). While the ability of several kinds of EPS to facilitate attachment to plant surfaces has been reported in various bacteria (Danhorn and Fuqua, 2007; Yaron and Römling, 2014; Castiblanco and Sundin, 2016), this is the first report describing such function of the *L. monocytogenes* Pss EPS.

Colonization efficiency depended on the properties of plant surface. The Pss EPS had the largest effect on rough, fiber-rich surfaces of wood and netted cantaloupe rind, and the smallest – on smooth surfaces of cantaloupe pulp (Figure 3B). More EPS-biofilms were formed on the edges than on the smooth surfaces of the same produce (Figure 3A). Because strains used in this study grow at the same rate, differences in colonization reflect differences in initial surface attachment, therefore Pss is involved in attachment to plant matter. While we did not directly interrogate the mechanism of Pss-mediated plant surface binding, the ability of Pss to bind to diverse plant matter, but not to manmade materials, and bind better to fiber-rich surfaces of wood and cantaloupe rind suggests that Pss interacts with the insoluble plant (ligno)cellulose fibers. Only few additional factors promoting attachment to plant matter are known in *L. monocytogenes*. One of these is functional flagella. They enhance initial attachment to plant surfaces in some *L. monocytogenes* strains (Lemon et al., 2007; Gorski et al., 2009). Another factor is a cellulose binding protein, Lcp (LMOf2365_0859). Strain *L. monocytogenes* F2365 attached to cantaloupe rind, lettuce, and spinach better than the *lcp* mutant by several-fold (Bae et al., 2013). Lmo0842, an Lcp homolog, is encoded in the EGD-e genome and may have the same function. Note, however, that voluminous Pss EPS synthesized by the Δ*pdeB/C/D* strain likely masked surface proteins, including Lmo0842. Because the EPS-synthesizing strain is impaired in motility (Chen et al., 2014), Pss also likely negated the effects of flagella.

In addition to enhancing colonization, the Pss EPS strongly protected *L. monocytogenes* grown in the EPS-biofilms on the surfaces of fruit and vegetables against desiccation and acid stresses. These stresses are arguably most relevant from the standpoint of fresh produce safety because they mimic, respectively, conditions of produce storage and transportation, and passage of the contaminated produce pieces through the stomach following consumption. In our experimental setup, EPS-biofilms showed a 6−16-fold increase in desiccation tolerance (Figure 5), and a 11−116-fold increase in acid stress survival (Figure 6). The findings that listerial EPS-biofilms are much more tolerant to stresses are in line with the role of EPS-biofilms in other bacteria (Mao et al., 2006; Deka et al., 2019). These findings are also consistent with our earlier observations that clumps of the Δ*pdeB/C/D* strain formed in liquid media are much more tolerant of desiccation and exposure to harmful chemicals, compared to planktonically grown cells (Chen et al., 2014).

To estimate the overall effect that the *L. monocytogenes* EPS on safety of such produce as cantaloupes, we combined the effects of Pss on produce colonization (Figures 1C, 3B, C; 2−12-fold increase in CFUs), ability to survive during storage and transportation (Figure 5, 6−16-fold increase in desiccation tolerance) and ability to survive stomach acid exposure (Figure 6, 11−116-fold increase in acid stress survival). By multiplying the low- and high-end values, we estimate that the EPS-synthesizing strain has an enormous, 10^2^- to 10^4^-fold, advantage over EPS-impaired strains in reaching the small intestine of consumers where they may cause disease. If desiccation is not part of processing, the advantage would remain very large. Note that these numbers may be underestimates, because the bulk (∼90%) of the EPS-biofilms was removed prior to the desiccation experiment, in order to bring pre-desiccation CFUs of all strains to the same range. Further, the 48-h biofilms tested in this study may not have fully developed protective potential, compared to older biofilms (Hingston et al., 2013; Chen et al., 2020). We surmise that the enormous advantage imparted by EPS to listeria on fresh produce makes EPS an extremely problematic factor from the standpoint of fresh produce safety.

The key question not addressed in this work is how often listerial EPS-biofilms are formed on plant matter, including fresh produce. On the one hand, *Listeria* are frequently associated with the decaying plant matter in nature (Welshimer and Donker-Voet, 1971; Fenlon, 1986; Ivanek et al., 2007; Freitag, 2009). The benefits rendered by Pss regarding enhanced colonization and stress tolerance point to the high likelihood that EPS is synthesized on plant matter. Further, the *pssA-E* operon encoding the Pss EPS biosynthetic machinery is part of the core *L. monocytogenes* genome, deciphered from 1,696 genomes of strains isolated from diverse sources and geographic locations (Moura et al., 2016). Strong conservation of the *pssA-E* operon in the genomes of pathogenic and non-pathogenic listeria supports the notion that Pss is important for environmental survival (Chen et al., 2014). On the other hand, the Pss EPS has been unambiguously detected only in the *L. monocytogenes* strains with artificially elevated c-di-GMP levels, either via mutations (e.g., Δ*pdeB/C/D*) or via expression of a foreign DGC (Chen et al., 2014). Under the conditions used in this study, the wild type, EGD-e, showed no solid evidence of Pss. If it was synthesized, the amounts were not readily detectable by SEM (Figure 4) and were low to make a major difference in colonization and stress survival, compared to the EPS-impaired strains (Figures 1C, 3, 5, 6). It is of course possible that EGD-e is not an optimal strain for investigating EPS synthesis, because it has been propagated under the laboratory conditions for several decades (Murray et al., 1926). Such maintenance is known to select against biofilm phenotypes resulting in domesticated strains (McLoon et al., 2011). *L. monocytogenes* strains isolated from the recent fresh produce outbreaks may therefore offer more relevant insights into EPS synthesis.

Additional factors that may have obscured EPS presence in *L. monocytogenes* biofilms concern experimental design of most studies of listerial attachment to plant matter. *L. monocytogenes* in these studies was usually grown in rich liquid media, spotted on the surfaces of fresh produce, and allowed to dry. Bacterial biofilms and survival were then investigated over time at different environmental conditions, such as temperature and humidity (Marik et al., 2020). This setup undervalues time needed for planktonic, single-cell bacteria from liquid medium to switch to the surface-attached, biofilm lifestyle, when EPS synthesis is expected to take place (Römling et al., 2013). Further, rich media used in those studies may not adequately mimic conditions under which listerial biofilms are formed on plant matter, whether in the environment or in fresh produce processing facilities. It is also important to note that the majority of listerial biofilm studies involved manmade materials used in food processing, such as stainless steel, aluminum, rubber, glass, and plastics (Blackman and Frank, 1996; Bonaventura et al., 2008; Lee et al., 2017). The extracellular biofilm matrices on these materials contain mostly eDNA and secreted proteins (Blackman and Frank, 1996; Rieu et al., 2008; Harmsen et al., 2010; Ferreira et al., 2014; Guilbaud et al., 2015; Zetzmann et al., 2015; Oloketuyi and Khan, 2017; Rodríguez-López et al., 2018), and biofilm forming capacity is highly dependent on specific strains and experimental conditions (Borucki et al., 2003; Cherifi et al., 2017; Lee et al., 2017; Gorski et al., 2021; Gray et al., 2021). The lack of EPS under these conditions is consistent with the results of this work that showed that Pss does not affect colonization of manmade materials (Chen et al., 2014; Figure 1).

To better understand the role of the *L. monocytogenes* Pss EPS in nature and in fresh produce industry, we need to uncover environmental factors that increase c-di-GMP levels and thus turn on Pss synthesis. Having a Pss-specific probe would also be helpful for detecting Pss EPS on plant matter. Work in these areas is ongoing, as is the search for compounds that inhibit formation of EPS-biofilms. Even if such biofilms are formed on fresh produce only by some strains and only under specific conditions, from the standpoint of fresh produce safety, the 10^2^−10^4^-fold advantage that EPS may render *L. monocytogenes* in causing foodborne illness seems too large to ignore.

## Materials and methods

### Bacterial strains, plasmids, and growth conditions

Strains and plasmids used in this study are listed in Table 1. The c-di-GMP null strain, Δ*pdeB/C/D* Δ*dgcA/B/C*, was constructed from strain Δ*pdeB/C/D* using sequential deletion of *dgcA/B* (with the help of pKSV7*-*Δ*dgcAB*) and *dgcC* (with the help of pKSV7-Δ*dgcC*), as described earlier (Chen et al., 2014). Plasmid pAM-mScarlet expressing the red fluorescent protein mScarlet (Bindels et al., 2017) was constructed to enhance visualization of *L. monocytogenes* on the surfaces of wood and fresh produce. The mScarlet gene was synthesized with *L. monocytogenes*-optimized codons (Twist Bioscience) and cloned downstream of the strong *Bacillus subtilis* Pveg promoter (Guiziou et al., 2016) in the pAM101-derived vector (Fujimoto and Ike, 2001). The listerial strains were grown in the liquid minimum HTM medium (Tsai and Hodgson, 2003) containing 3% glucose and 0.2% yeast extract, HTM/GY, at 30°C under shaking (220 rpm). When strains containing pAM-mScarlet were used, the media was supplemented with 10 μg/mL chloramphenicol (Cm). For enumerating CFUs, cultures were routinely plated onto Brain Heart Infusion (BHI) agar (Millipore Sigma, Burlington, MA, United States) and incubated at 37°C for 36 h.

**TABLE 1:** Strains and plasmids used in this study.

Strain and plasmid	Relevant genotype or description	References or source
**Strains**		
*Escherichia coli*		
DH10β	Strain used for plasmid maintenance	New England BioLabs
*Listeria monocytogenes*		
EGD-e	Wild type	ATCC BAA-679*
Δ*pdeB/C/D*	EGD-e containing deletions in the *pdeB, pdeC* and *pdeD* genes	Chen et al., 2014
Δ*pdeB/C/D*Δ*pssC*	Δ*pdeB/C/D* and in-frame deletion in *pssC*	Köseoğlu et al., 2015
Δ*pdeB/C/D*Δ*dgcA/B/C*	c-di-GMP null mutant	This study
**Plasmids**		
pAM-mScarlet	pAM101-derived vector containing the mScarlet gene under the P*_*veg*_* promoter, Cm^r^	This study
pKSV7-Δ*dgcA/B*	Plasmid for in-frame deletion of *dgcAB*	Chen et al., 2014
pKSV7-Δ*dgcC*	Plasmid for in-frame deletion of *dgcC*	Chen et al., 2014

*ATCC, American Type Culture Collection.

### Preparation and treatment of coupons used in biofilm experiments

All coupons (disks for do-it-yourself arts and crafts projects) were purchased on from various vendors, including coupons made from unspecified wood (32 mm diameter × 1.2 mm thickness, Woodpile), birch (50 mm × 8 mm, Woodpeckers), poplar (50 mm × 2.5 mm, Juvale), *Schima superba* (38 mm × 4 mm, Axe Sickle), stainless steel (38 × 1.55 mm, PH Pandahall, CA, USA), aluminum (38 × 1.55 mm, RPM Stamping Blanks, Rose Metal Products, Springfield, MO, USA), and acrylic (25.4 × 1.8 mm, Tupalizy, PTC-Office). Prior to use, all non-wood coupons were immersed in 70% ethanol for 20 min for disinfection, rinsed twice in sterile deionized water and air dried. The wood coupons were autoclaved (121°C, 30 min). In some experiments, prior to sterilization, surfaces of steel, aluminum and acrylic coupons were roughened up by a scrapper to imitate surface roughness of wood coupons.

The overnight *L. monocytogenes* cultures were diluted 1:100 into 10 mL HTM/GY medium in 125-mL flasks and grown at 30°C until optical density of ∼ 0.4 at 600 nm. Sterile wood coupons or pieces of fresh produce were added at this point and cultivation was continued for 48 h. The coupons were subsequently withdrawn and rinsed twice in sterile HTM to remove loosely attached biofilms. The biomass attached to the coupons and produce pieces was thoroughly scrapped off into HTM medium using a sterile scalpel, and the suspensions were vortexed. Serial dilutions were plated onto BHI agar plates and grown at 37°C for 36 h followed by CFU enumeration.

### Preparation and inoculation of pieces of fresh produce

Whole cantaloupes, celery, and mixed salads (Iceberg lettuce, red cabbage, carrots) used in this study were purchased from local (Laramie, WY) retail stores. Cantaloupe coupons (20 mm × 4 mm) were obtained by using a cork borer. Some pieces were cut out to contain rind, others were cut out to contain cantaloupe pulp only. Celery was cut in pieces of varying lengths from similar sized stocks. Prior to processing, fresh produce was thoroughly washed. Cutout pieces were sterilized by complete submersion into 8.25% sodium hypochlorite (CloroxPro, Clorox Professional Products Company) in the 50-mL centrifuge tubes placed on a rocker platform for 20 min. Following sterilization, fresh produce pieces were submerged in 50-mL sterile distilled water overnight and subsequently rinsed in fresh sterile distilled water. Sterile pieces were added to listerial cultures, as described above for coupons.

Following 48-h incubation, produce pieces were aseptically removed from the cultures and rinsed by dipping in three sequential beakers with sterile HTM media. To measure listerial colonization, cantaloupe rind was carefully separated from pulp and processed separately, while the remaining pulp was discarded. To measure colonization of cantaloupe pulp, pulp-only pieces lacking rind were used. The produce pieces were mechanically macerated in the Stomacher bags in 5 mL HTM medium and homogenized for 10 min at high speed (Stomacher^®^ 80 Biomaster, Seward, UK). The serial dilutions of the homogenates were plated onto BHI agar plates for CFU enumeration.

### SEM analysis of listeria-plant biofilms

Small (5 mm diameter x 1 mm thickness) pieces of cantaloupe rind containing *L. monocytogenes* biofilms were used for SEM (Scanning Electron Microscope FEI Quanta 250). Briefly, rind pieces were fixed for 2 h in 2% glutaraldehyde-PBS buffer at room temperature, followed by dehydration steps in a series of ethanol baths (10 min each) containing 25, 50, 75, 95, and 100% (3 times) ethanol, and postfixed by incubation with 1% osmium tetroxide in PBS for 1 h in the dark. The samples were then dried using Balzers Critical Point Dryer (CPD 020) before coating with gold-palladium, as described earlier (Muravnik et al., 2016).

### Desiccation survival of *L. monocytogenes* in biofilms on cantaloupe rind

Rind-containing cantaloupe pieces were removed from the liquid *L. monocytogenes* cultures after 48-h incubation. To adjust biomass levels to approximately the same initial cell numbers, the voluminous EPS-biofilms of the Δ*pdeB/C/D* strain were gently brushed off cantaloupe rind, resulting in the removal of ∼ 90% biomass. Coupons were then aseptically cut into four equal-sized quarters. One quarter was used to determine initial CFU (day 0) following homogenization in Stomacher. The remaining quarters were placed on sterile blotting paper in open Petri dishes and kept at room temperature inside the biological safety cabinet with the switched off blower. After 7, 14, and 21 days of storage the quarters were placed in Stomacher bags with 5 mL HTM medium, soaked for 1 h, homogenized and serial dilutions were plated for CFU enumeration.

### Acid stress survival of *L. monocytogenes* in biofilms on celery pieces

Biofilms formed on the pieces of celery and cantaloupe pulp were used in acid stress experiments. The sizes of celery pieces were adjusted to have similar initial number of listeria in biofilms, as follows: 20 mm (EGD-e), 5 mm (Δ*pdeB/C/D*), 22 mm (Δ*pdeB/C/D*Δ*pssC*), and 24 mm (Δ*pdeB/C/D*Δ*dgcA/B/C*). The 4-mm thick cantaloupe pulp pieces had the following diameters: 18 mm (EGD-e), 15 mm (Δ*pdeB/C/D*), 19 mm (Δ*pdeB/C/D*Δ*pssC*), and 20 mm (Δ*pdeB/C/D*Δ*dgcA/B/C*). Following 48-h incubation in liquid *L. monocytogenes* cultures, celery or cantaloupe pulp pieces were removed and placed in HTM/GY acidified to pH 5.0 or 2.5 with hydrochloric acid for 15 min at 37°C. A preconditioning protocol involved exposing celery pieces to pH 5.0 for 15 min followed by exposure to pH 2.5 for additional 15 min, as described (Guerreiro et al., 2022). The acid was neutralized to pH 7.0 by addition of the sodium hydroxide solution, celery pieces were homogenized, and serial dilutions were plated for CFU enumeration.

## Statistical analysis

Microsoft Excel was used for data processing analysis and presentation. The bar charts display a mean ± standard deviation (SD) from three independent experiments, each of which had at least two replicates. Unpaired Student’s *t*-tests were performed using Prism 9 for Mac (GraphPad). Significant differences, compared to the wild type, EGD-e, are indicated in all figures as follows: *, *P* < 0.05; ^**^, *P* < 0.01; ^***^, *P* < 0.001, and ^****^, *P* < 0.0001.

## Data availability statement

The raw data supporting the conclusions of this article will be made available by the authors, without undue reservation.

## Author contributions

AF performed the experiments, analyzed the data, and wrote the manuscript. AE performed the experiments and analyzed the data. L-HC constructed the *L. monocytogenes* c-di-GMP null strain. MG conceived, designed, coordinated the research, and wrote the manuscript. All authors contributed to the article and approved the submitted version.
